# Irradiation-Induced *Deinococcus radiodurans* Genome Fragmentation Triggers Transposition of a Single Resident Insertion Sequence

**DOI:** 10.1371/journal.pgen.1000799

**Published:** 2010-01-15

**Authors:** Cécile Pasternak, Bao Ton-Hoang, Geneviève Coste, Adriana Bailone, Michael Chandler, Suzanne Sommer

**Affiliations:** 1 Université Paris-Sud, Centre National de Recherche Scientifique, Unité Mixte de Recherche 8621, LRC CEA 42V, Institut de Génétique et Microbiologie, Bât. 409, Orsay, France; 2 Laboratoire de Microbiologie et Génétique Moléculaires, Centre National de Recherche Scientifique, Unité Mixte de Recherche 5100, Toulouse, France; Stanford University, United States of America

## Abstract

Stress-induced transposition is an attractive notion since it is potentially important in creating diversity to facilitate adaptation of the host to severe environmental conditions. One common major stress is radiation-induced DNA damage. *Deinococcus radiodurans* has an exceptional ability to withstand the lethal effects of DNA–damaging agents (ionizing radiation, UV light, and desiccation). High radiation levels result in genome fragmentation and reassembly in a process which generates significant amounts of single-stranded DNA. This capacity of *D. radiodurans* to withstand irradiation raises important questions concerning its response to radiation-induced mutagenic lesions. A recent study analyzed the mutational profile in the *thyA* gene following irradiation. The majority of *thyA* mutants resulted from transposition of one particular Insertion Sequence (IS), IS*Dra2*, of the many different ISs in the *D. radiodurans genome*. IS*Dra2* is a member of a newly recognised class of ISs, the IS*200*/IS*605* family of insertion sequences.

## Author Summary

Induction of transposition in prokaryotes under cell stress conditions is potentially important in creating diversity facilitating adaptation to severe environments. In *Deinococcus radiodurans*, the most radiation-resistant organism known, despite abundance of resident insertion sequences (IS), transposition of a single IS, IS*Dra2*, was found to be strongly induced by irradiation. We show that both steps involved in transposition, IS excision, and insertion, increase significantly following host cell irradiation and, using PCR analysis of genomic DNA, that exposure to γ-irradiation stimulates massive excision of the single genomic IS*Dra2* copy as a DNA circle and reclosure of the empty site. These events are closely correlated with the initiation of the process leading to genome reassembly from chromosomal fragments, which occurs mainly through a mechanism generating long stretches of single-stranded DNA. Consistent with this, we also demonstrate a requirement for single strand DNA substrates in TnpA-catalysed cleavage and strand transfer *in vitro* . Since we find no evidence for irradiation-induced expression of the IS*Dra2* transposase, we infer that transposition is triggered by the increase in its single-strand DNA substrate. The potential impact on genome reassembly and in creating genome host diversity by triggering transposition in this way is discussed.

## Introduction

Stress-induced transposition has been an attractive notion for some time since it is potentially important in creating diversity to facilitate adaptation of the host to severe environmental conditions. One common major stress is DNA damage. This induces a variety of responses including changes in expression of numerous genes [1]–[4], cell cycle arrest [5],[6], induction of bacterial prophages [7]–[8] and, by generating diversity, can also aid development of processes such as bacterial pathogenicity and virulence [9].

Several studies have focused on DNA damage-induced transposition in bacteria but have not yet provided a coherent mechanistic scenario. This interest presumably stemmed directly from capacity of UV-irradiation to promote lysogenic induction [7]. Indeed, although IS*10* transposition was shown to be induced by UV light in an SOS-dependent pathway [10], the precise mechanism has not been elucidated. A complex relationship between the SOS response and Tn*5* transposition has emerged from contradictory reports [11]–[12]. More recently, activation of Tn*7* transposition into regional hotspots by double-strand breaks, has suggested a relationship between Tn*7* transposition and DNA repair [13], but direct evidence is still missing. Finally, numerous host factors can modulate transposition in *E. coli* in response to stress [14], but their specific roles are presently unknown. Here we identify and demonstrate the molecular basis of a strong radiation-stimulated response of transposition in the irradiation resistant *Deinococcus radiodurans*.

*D. radiodurans* has an exceptional ability to withstand the lethal effects of DNA-damaging agents, such as ionizing radiation, UV light and desiccation (for reviews, see [15],[16],[17]). High radiation levels result in genome fragmentation and reassembly in a process which generates significant amounts of single stranded (ss) DNA [18]. In addition to this extraordinary ability to reassemble its genome, the capacity of *D. radiodurans* to withstand irradiation also raises important questions about the mechanisms involved in the response to and repair of radiation-induced mutagenic lesions.

A recent study analysed the mutational profile in the *thyA* gene following doses of 10 kGy of γ- or 600 J m^−2^ of UV-irradiation. The majority of *thyA* mutants were due to a single insertion of one particular IS belonging to the IS*200*/IS*605* family: IS*Dra2* [19](originally named IS*8301* [20]). While some mutants, presumably resulting from point mutations or small insertions or deletions, retained the length of the wild-type gene, the many other resident ISs unrelated to IS*Dra2* made only small contributions to the mutant pool despite their presence in significant numbers (see www-IS.biotoul.fr) in the *D. radiodurans* R1 genome sequence [21]. The importance of the contribution of IS*Dra2* to mutagenesis is further underlined by its low genomic copy number in the standard R1 ATCC 13939 strain used in our studies as judged by a combination of whole genome hybridization and sequencing [19]: 1 complete and 1 inactive degenerate IS*Dra2* copy (in contrast to the published *D. radiodurans* genome sequence which revealed 7 complete and one partial IS*Dra2* copy [21]). Since another member of the IS*200*/IS*605* family, IS*608* from *Helicobacter pylori*, uses obligatory ssDNA intermediates [22], it seemed possible that the signal which triggers IS*Dra2* transposition is the very event which leads to genome reassembly: the formation of ssDNA.

**Figure 1:**
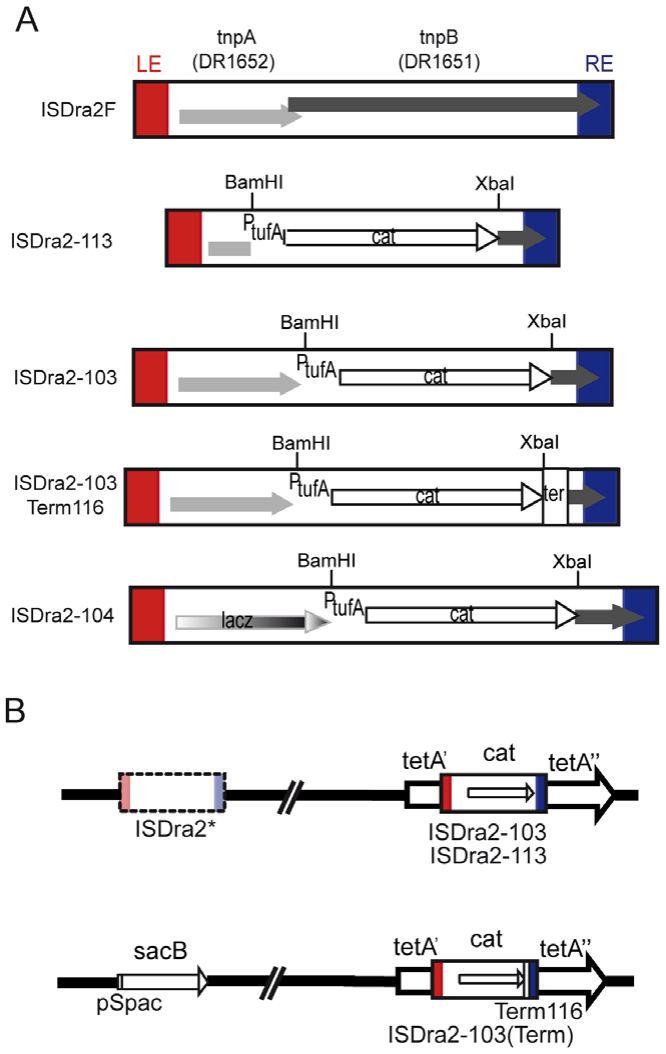
A genetic assay for IS*Dra2* transposition. (A) Derivatives of IS*Dra2* (1736 bp). Orfs are indicated as boxes with arrowheads showing the direction of translation; LE and RE, red and blue boxes, respectively. In IS*Dra2*-113 (1509 bp) both *tnpA* and *tnpB* were replaced by a Cam^R^ cassette, while IS*Dra2*-103 (1778 bp) was deleted only of *tnpB* and expresses *tnpA* from its natural promoter. IS*Dra2*-104 results from replacement of the *tnpA* coding region of IS*Dra2*-103 by the *lacZ* coding region. IS*Dra2*-103Term116 (1889 bp) carries the Deinococcal transcription terminator Term116 [45] downstream of the *cat* gene. (B) *In vivo* genetic assay to measure excision and insertion events of a derivative of IS*Dra2*. The IS*Dra2*F copy was first replaced by a Tet^R^ cassette and IS*Dra2*-113 (or IS*Dra2*-103) was inserted at the unique TTGAT target site present in the *tetA* gene. The second inactive copy, ISDra2*, was replaced by the *sacB* gene and an accompanying hygromycin (hyg) resistance cassette as a selective marker. TnpA-dependent IS*Dra2* excision restores a functional *tetA* gene, giving rise to Tet^R^ colonies while insertion into the reporter *sacB* gene confers resistance to sucrose. See Figure S1 and its legend.

We have explored the properties and behaviour of IS*Dra2* (IS*Dra2*F; Figure 1A) in *D. radiodurans* and have identified the mechanism by which IS*Dra2* transposition is triggered by radiation. Using a genetic system to detect two principal transposition steps, transposon excision and insertion, we show that IS*Dra2*, like other IS*200*/*605* family members, requires TnpA but not TnpB for both and that insertion occurs 3′ to a specific pentanucleotide (as deduced from genome analyses [20]). We demonstrate genetically that both steps are significantly increased following host cell irradiation. We also show that the entire TnpA-catalysed transposition cycle including excision and insertion depends strictly on single strand DNA substrates *in vitro*. Finally, using a PCR-based approach, we demonstrate that, *in vivo*, exposure to γ-irradiation stimulates excision of the single genomic copy of IS*Dra2* from the genome in the form of a DNA circle. These events are closely correlated with the initiation of the process leading to genome reassembly from chromosomal fragments, which occurs mainly through a mechanism generating long stretches of single stranded DNA [18].

## Results

### IS*Dra2* excision and insertion *in vivo* in *D. radiodurans*

IS*200*/IS*605* family members often carry 2 genes, *tnpA*, encoding the transposase and *tnpB*, a gene of unknown function (Figure 1A). As shown for the related IS608 [23], the transposition cycle occurs in two principal steps: excision from the donor backbone in the form of a single strand circle and subsequent insertion into a suitable DNA target [22]. These steps were monitored for IS*Dra2* transposition in *D. radiodurans* using a genetic assay. TnpA and/or TnpB were expressed *in trans* from a plasmid, pGY11559, under control of the IPTG-inducible P*_spac_* promoter [19] (Table S1). For excision, the unique active IS*Dra2* copy (IS*Dra2*F: *D. radiodurans* loci *DR1651*-*DR1652*) was replaced either by a derivative, IS*Dra2*-113, retaining functional IS ends but in which *tnpA* and *tnpB* were replaced by a Cam^R^ cassette, or by IS*Dra2*-103, similar to IS*Dra2*-113 but retaining *tnpA* controlled by its own promoter (Figure 1A). The resident IS*Dra2F* was first replaced by a Tet^R^ cassette and IS*Dra2*-113 (or IS*Dra2*-103) was inserted by homologous recombination at the unique 5′TTGAT3′ target site (see below) present in the *tetA* gene. IS excision restores a Tet^R^ phenotype (Figure 1B). These constructions are described in detail in Figure S1.

Ectopic TnpA expression in this system induced IS*Dra2*-113 excision at a frequency of 2×10^−3^ (Table 1). This was not increased when IPTG was added to the medium, to induce TnpA expression. Similar experiments in which P*_spac_* was used to drive a *lacZ* gene in plasmid pGY11556 (Figure S2) showed that addition of IPTG resulted in a 143-fold increase in β-galactosidase activity. This suggests that sufficient transposase would be produced by escape synthesis from P*_spac_* to ensure transposition and that activity is limited by the supply of a correct DNA substrate. Activity obtained with ectopic TnpB alone (<1×10^−9^) was similar to the background levels observed with the empty vector plasmid (<4.8 10^−9^) clearly demonstrating that TnpA is absolutely required for IS*Dra2* excision while TnpB is dispensable. Similar results were obtained with IS*Dra2*-103 (Figure 1A), in which *tnpA* was in its natural location in the IS sequence and expressed from its own promoter (Table 1).

**Table 1:** Excision frequencies of IS*Dra2* derivatives.

IS*Dra2* derivative	Plasmid	Inducing treatment	Excision frequency (Tet^R^/viable cell) (a)
IS*Dra2*-113: Δ(*tnpA tnpB*)Ω*cat*	pGY11559 (empty)	None	<4.8 10^−9^
	pGY13203 (*tnpA*)	None	2 (±0.6) 10^−3^
		γ-rays (b)	2.7 (±0.4) 10^−2^
		UV (c)	3.22 (±0.89) 10^−2^
	pGY13204 (*tnpB*)	None	<1 10^−9^
IS*Dra2*-103: *tnpA^+^*Δ*tnpB* Ω*cat*	None	None	2.62 (±2.2) 10^−3^
		γ-rays (b)	2.69 (±0.17) 10^−2^
		UV (c)	1.52 (±0.37) 10^−2^
IS*Dra2*-103Term116: *tnpA^+^*Δ*tnpB* Ω*cat*::Term116	None	None	2.32 (±0.9) 10^−3^
		γ-rays (b)	1.85 (±0.3) 10^−2^

(a) The excision frequency of IS*Dra2* derivatives was calculated as described in Materials and Methods.

(b) 5 kGy.

(c) 600 J m^−2^.

To measure transposon insertion, both non-targeted and targeted approaches were used. In both cases, we measured insertion in the subpopulation of cells in which the transposon IS*Dra2*-113 had excised from its initial locus and had inserted elsewhere in the genome as judged respectively by the reconstitution of the Tet^R^ gene and retention of the Cam^R^ marker carried by the IS derivative.

In the non-targeted approach, the proportion of Cam^R^ among Tet^R^ clones, reflecting the frequency of spontaneous insertion, was about 10^−2^ in strain GY13120 (Figure 1B, top) expressing TnpA ectopically, compared to <10^−9^ in the absence of TnpA. These insertions were true transposition events since they had occurred 3′ to the pentanucleotide, 5′TTGAT3′ (Table S2), the sequence preceding the left end of IS*Dra2* in all genomic loci identified [20].

In the targeted approach, the degenerate IS*Dra2* copy (ISDra2* loci *DR0177*-*DR0178*) was replaced with the target *sacB* gene from *Bacillus subtilis*, coupled to a hygromycin resistance cassette to assist the construction (Figure 1B, bottom, and Figure S1). As in other bacteria [24],[25], *sacB* expression in the presence of sucrose is lethal for *D. radiodurans* (data not shown) and inactivation of *sacB*, for example, by IS insertion, confers a sucrose resistant phenotype. Moreover, *sacB* contains 10 copies of the insertion site 5′TTGAT3′ (9 on one strand and 1 on the complementary strand; Figure S3A). For this analysis, it was considered prudent to include a transcriptional terminator (Term116) downstream of the *cat* gene of IS*Dra2*-113 to avoid possible interference of *sacB* transcription from the strong P*_spac_* promoter with expression of the *cat* gene (Figure 1A and 1B; Figure S1). By imposing a triple selection for Tet^R^, Cam^R^ and Suc^R^, we were able to directly collect clones in which IS*Dra2*-103Term116 had excised from its resident site (Tet^R^) and inserted into *sacB* (Suc^R^). The nucleotide sequence of the Suc^R^ mutants confirmed that each had IS*Dra2*-113 inserted into one of the 5′TTGAT3′ target sequences (Figure S3A and Figure 3B).

Together, these results demonstrate that TnpA alone is sufficient for both transposon excision and insertion.

### Effect of UV and γ-radiation on excision and insertion

Using this genetic system, we then measured the excision frequency of IS*Dra2*-113 and IS*Dra2*-103 following exposure to UV- (600 J m^−2^) or γ-irradiation (5 kGy). Both treatments increased excision frequencies about 10-fold (Table 1).

To determine whether γ-ray irradiation also stimulates the insertion step of transposition, we measured the insertion frequency of IS*Dra2*-103 (Term116) expressing TnpA from its own promoter (to remain as close to natural conditions as possible) (Figure 1A) into *sacB*. The frequency of Tet^R^ colonies and of Suc^R^ Cam^R^ Tet^R^ colonies in cells irradiated with 5 kGy of γ-rays and in non-irradiated cultures was measured in the tester strain GY13174 carrying IS*Dra2*-103 (Term116). The Tet^R^ frequency, which monitors the excision step, rose from 2.32×10^−3^ to 1.85×10^−2^ after γ- irradiation (Table 1; average values of 10 independent experiments). The frequency of Suc^R^ Cam^R^ Tet^R^ colonies, representing the overall transposition frequency into the reporter gene, also increased from 3.76×10^−10^ to 1.8×10^−8^ after γ- irradiation (Table 2).

**Table 2:** Insertion frequencies of IS*Dra2*-103Term116 into *sacB*.

IS*Dra2* derivative	Plasmid	Inducing treatment	Tet^R^ Cam^R^ Suc^R^/viable cell	Tet^R^ Cam^R^ Suc^R^/Tet^R^	Insertion stimulation factor
IS*Dra2*-103Term116: *tnpA^+^*Δ*tnpB* Ω*cat*::Term116	None	None	3.76 (±0.52) 10^−10^	1.68 (±0.54) 10^−7^	1
		γ-rays (b)	1.8 (±0.75) 10^−8^	1 (±0.49) 10^−6^	5.95

(b) 5 kGy.

Thus, both excision and insertion of the IS*Dra2* derivatives were stimulated by irradiation. This stimulation is unlikely to result from an increase in *tnpA* expression after irradiation since strain GY14310 in which the coding region of *tnpA* was replaced by the coding region of *lacZ* in IS*Dra2*-104 (Figure 1A) showed no detectable increase in β-galactosidase activity at 0, 30, 60, 120 or 180 min post-irradiation (Figure S2 and data not shown).

### *In vitro* cleavage, strand transfer, and insertion of IS*Dra2*

Since the related TnpA from IS*608* uses obligatory single-stranded DNA substrates in *in vitro* transposition reactions [22], we suspected that the stimulation of IS*Dra2* excision and insertion might be linked to the formation of single-stranded DNA during repair of DNA damage which would supply the appropriate substrate.

To confirm that IS*Dra2* TnpA is active only on single-strand DNA substrates, we used an *in vitro* system, developed for the related IS*608*, to investigate TnpA-catalysed cleavage and strand transfer [22],[26]. IS*608* transposition reactions are strand specific and use, by definition, the top strand. Recombination reactions recapitulating transposon excision and donor joint formation were performed. For this, the IS*Dra2 tnpA* gene was cloned with a C-terminal His_6_ tag under control of a p*_lac_* promoter (Materials and Methods) and the protein was purified as described previously [23]. The C-terminal His-tagged TnpA from *D. radiodurans* was active since it catalysed IS*Dra2*-113 excision *in vivo* in a tester strain expressing TnpA-His6 under the control of P*_spac_* promoter in plasmid pGY13505 (data not shown).

**Figure 2:**
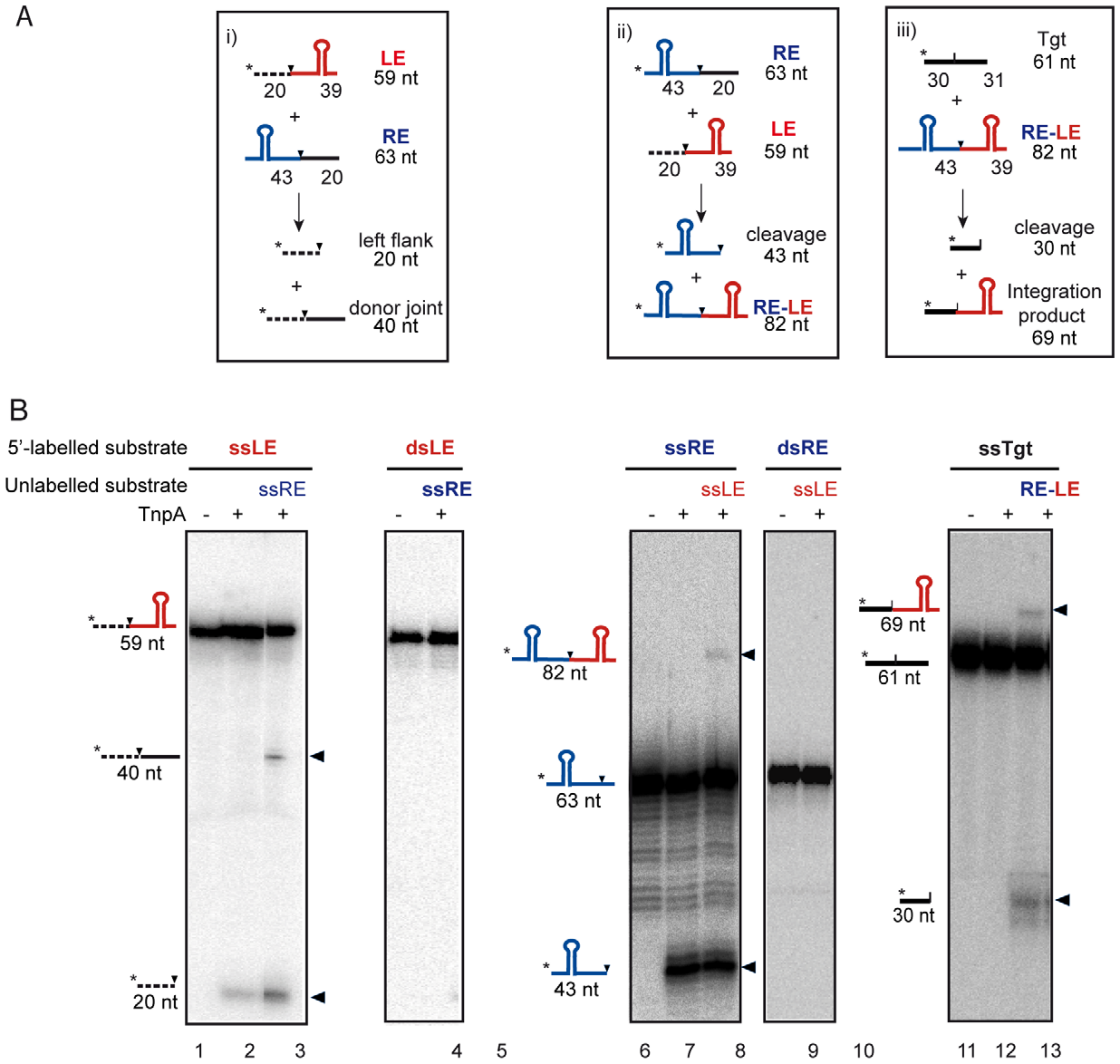
IS*Dra2* TnpA-catalyzed cleavage and strand transfer *in vitro*. (A) Oligonucleotides used as DNA substrates. Length of cleavage products is indicated. The potential secondary structure in both LE and RE is indicated. Black dotted and black lines: left and right DNA flanks cleavage sites are shown as vertical black arrows. Asterisk (*) indicates radioisotope position. (B) Excision *in vitro*: donor joint formation and single-versus double-strand substrates. The 5′-^32^P-labelled oligonucleotide used was the 59-base LE composed of 39 nt of LE and 20 nt 5′ to the 5′TTGAT3′ and the unlabelled 63-base oligonucleotide RE. Lane 1: no-protein control; lane 2: TnpA alone; lane 3: TnpA and unlabelled RE; lane 4: dsLE, no-protein; lane 5: dsLE, TnpA and ssRE.

The DNA substrate was a 59 nt single strand DNA fragment including the first 39 nt of IS*Dra2* LE carrying a subterminal secondary structure which serves as the TnpA binding site (Burgess-Hickman et al., in prep) and a 20 nt 5′ flank with the conserved pentanucleotide target sequence (5′TTGAT3′) (Figure 2A). Incubation of the 5′ end-labelled fragment (Figure 2B, lane 1) with purified TnpA in the presence of Mg^2+^ generated a cleaved 5′ end-labelled donor flank fragment of 20 nt (Figure 2B, lane 2). When mixed with an unlabelled 63 nt ss DNA fragment composed of the terminal 43 nt of RE (including the secondary structure) and a 3′ 20 nt flank, an additional fragment of 40 nt representing the joined donor flanks was generated (Figure 2B, lane 3). In contrast, double stranded LE (Figure 2B, lane 4) was neither cleaved nor underwent a strand transfer reaction with the unlabelled RE (Figure 2B, lane 5).

Similar results were obtained using 5′ end-labelled RE and unlabelled LE: cleavage and strand transfer were strictly dependent on ss subtrates (Figure 2B). We were also able to recapitulate the integration reaction *in vitro* using an end-labelled target DNA and an unlabelled RE-LE junction (Figure 2B).

Thus, the IS*Dra2* transposase is active on single- but not double-stranded substrates and is capable of cleavage of both LE and RE and of strand transfer to generate the donor joint and the RE-LE junction.

### Kinetics of irradiation-induced IS*Dra2* excision

**Figure 3:**
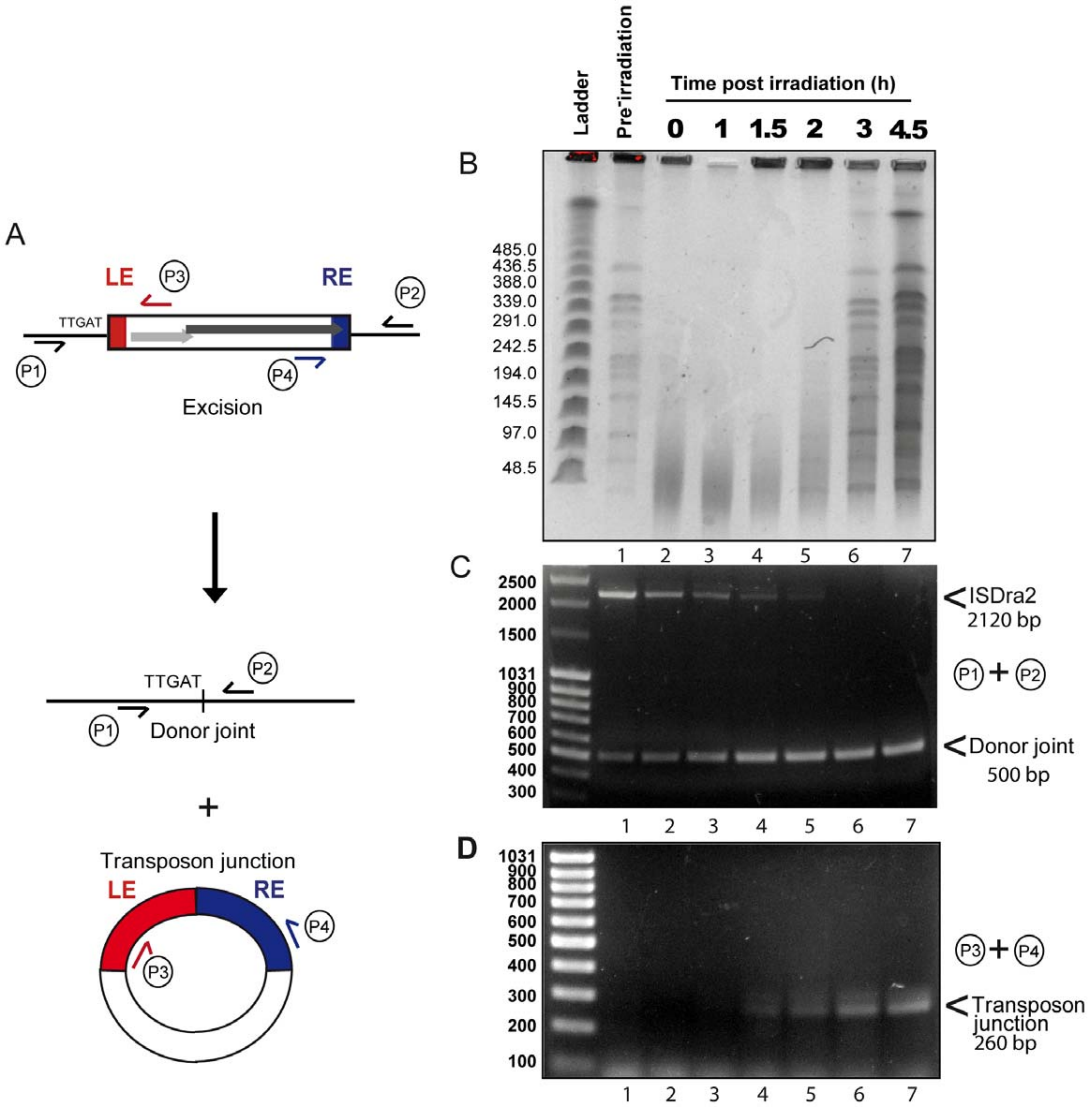
Physical evidence for irradiation-induced IS*Dra2* excision. Strain GY13120 (*tetA*ΩIS*Dra2*-113 expressing *tnpA in trans*) received 5 kGy of γ–irradiation and aliquots were taken to isolate genomic DNA used to prepare DNA agarose plugs and as template for PCR analysis. The time following irradiation is shown in hours above each lane. (A) Schematic representation of IS excision products. The excision products are shown together with the position of primers used in PCR analysis. Note that the transposon circle including the transposon junction is single stranded. (B) Kinetics of double-strand-break repair. DNA agarose plugs were digested with *Not*I prior to PFGE analyses and loaded onto a 1.06% agarose gel. L: λ Ladder. (C) Kinetics of donor joint appearance. PCR reactions were performed with primer pair P1 and P2 and loaded onto a 1% agarose gel; L: MassRuler DNA Ladder. (D) Kinetics of IS circle junction appearance. PCR reactions were performed with primer pair P3 and P4 and loaded onto a 2% agarose gel; L: O'GeneRuler 100 bp DNA Ladder.

To investigate the relationship between irradiation and induction of IS*Dra2* transposition, we analysed the kinetics of γ-irradiation-triggered IS*Dra2*-113 excision directly from the *D. radiodurans* chromosome. For this, we isolated genomic DNA before and at different times after γ-irradiation and subjected samples to PFGE analysis following *Not*I digestion (Figure 3B) and to PCR analysis (Figure 3A, 3C and 3D). Primer pairs P1+P2, complementary to the *tetA* flanks of IS*Dra2*-113 (Figure 3A), should generate a 2120 bp fragment when IS*Dra2*-113 is inserted into *tetA* and a 500 bp fragment when the donor backbone is sealed following IS*Dra2*-113 excision. IS circle junction formation was monitored using primers P3+P4, complementary to the subterminal IS region (Figure 3A), by the appearance of a 260 bp product representing abutted LE and RE.

Total *Not*I-digested genomic DNA (Figure 3B) showed complete fragmentation immediately following irradiation (compare lanes 1 and 2). Reassembly, evidenced by the gradual reappearance of distinct *Not*I fragments, could be detected at about 2 h post-irradiation while the complete regenerated banding pattern exhibited by the non-irradiated sample (lane 1) occurred after 3 h (lane 6). PCR analysis of these samples using the P1+P2 primer pair revealed the presence of the full length IS together with a low but significant quantity of donor joint prior to irradiation resulting from rejoining of the DNA flanks following IS excision (Figure 3C, lane 1). This indicates that IS excision occurs during normal growth of the host strain as might be expected as *tnpA* expression is under control of P*_spac_*. Importantly, a significant increase in the level of the donor joint (Figure 3C) occurred over the 4.5 h post-irradiation period. We note that the progressive disappearance of the full length IS does not necessarily reflect its excision. In order to visualize the donor joint product, the PCR conditions were adjusted (short extension times; see Materials and Methods) to favour amplification of shorter donor joint fragment. It is probable that the reduction in the intensity of full length IS is due to competition in the reaction mixture from the increasing concentration of the short donor joint. In addition, the P3+P4 primer pair revealed the gradual appearance between 1 and 1.5 h post irradiation of the IS junction species (Figure 3D). The identity of the donor joint and the IS junction was verified by sequencing. The IS junction generated by P3+P4 was cloned into a plasmid carrying Cam^R^ (unable to replicate in *D. radiodurans*) which was then introduced by transformation into a strain expressing ectopic TnpA. Ten independent Cam^R^ clones were analysed using inverse PCR and each was shown to carry an insertion 3′ to a 5′TTGAT3′ target sequence, demonstrating that the IS junction generated by P3+P4 is active in transposition (data not shown).

Although we did not use quantitative PCR, care was taken to include identical quantities of DNA in each reaction and, to a first approximation, the results indicate that both the donor joint and the transposon junction were formed with very similar kinetics. Products started to accumulate at a time which coincided with the end of DNA degradation and probably with the start of the ESDSA pathway generating long stretches of single stranded DNA [18]. These results therefore strongly suggest that the factor which triggers IS*Dra2* transposition is the formation of single stranded DNA during *D. radiodurans* genome assembly.

## Discussion

*D. radiodurans* has been the object of much interest due to its astonishing capacity to resist high levels of radiation [15],[17] and to the genome fragmentation and reassembly processes essential for its survival after irradiation [18]. Clearly such exceptional properties might influence the behavior of mobile genetic elements within the genome and perhaps reveal new and interesting regulatory mechanisms. One hint that this might be the case came from the observation that transposition of one IS, IS*Dra2*, into the *thyA* gene was apparently increased by high levels of γ- or UV-irradiation [19]. IS*Dra2* was the only insertion sequence of the 12 different IS family members present in the *D. radiodurans* genome to behave in this way.

We show that IS*Dra2* transposition is specifically triggered during the process of reassembly of the *D. radiodurans* genome which is associated with recovery from irradiation damage. IS*Dra2* belongs to the IS*200*/IS*605* family (see www-is.biotoul.fr) and the paradigm of this family, IS*608*, transposes by excision of a single strand circular DNA intermediate which can then insert into a single strand DNA target [22]. Our results demonstrate that IS*Dra2* also exhibits a strict requirement for single stranded DNA *in vitro* (Figure 2). Furthermore, we show genetically that both IS*Dra2* excision (detected by the restoration of an intact *tetA* gene) and insertion (into the reporter *sacB* resulting in resistance to sucrose) require TnpA, and that insertion occurs 3′ to the specific pentanucleotide sequence, 5′TTGAT3′, found adjacent to the left end of all naturally occurring genomic IS*Dra2* copies [20]. Moreover, we observe a 50 to 60-fold increase in the overall frequency of transposition following UV or γ-irradiation resulting from stimulation of the two transposition steps: a 8-fold increase in excision and a 6-fold increase in insertion of the IS circle transposition intermediate (Table 1). The overall transposition stimulation factor is in accord with the 50- and 100-fold induction of *in vivo* transposition of wild-type IS*Dra2* into the *thyA* gene by UV- and γ-irradiation respectively [19].

Monitoring *tnpA* promoter activity with the *lacZ* reporter gene demonstrated that the radiation-triggered IS*Dra2* transposition was not due to a specific induction of TnpA expression. Moreover, a same stimulatory effect of irradiation was observed whether TnpA was expressed from its natural promoter or from an external IPTG-inducible P*_spac_* promoter.

Importantly, using a physical PCR-based approach, we also demonstrate that IS*Dra2* excision, reclosure of the chromosomal DNA donor joint and formation of a transposon joint with abutted left and right ends (consistent with a circular form of IS*Dra2*) occur within irradiated *D. radiodurans* cells. Both rejoined donor DNA flanks and the LE-RE junction begin to accumulate after 90 min of post-irradiation incubation. This correlates with the end of the degradation of damaged DNA and the start of DNA double strand break repair processes such as extended synthesis dependent strand annealing (ESDSA) that generates high concentration of single stranded DNA [18],[27].

Induction of transposition by exposure to environmental stress has often been tacitly assumed. However, there has been only limited supporting evidence for the idea that transposition can be induced efficiently by environmental insults and the sparse data available are generally based on indirect genetic assays [10], [12]–[13], [28]–[32]. These data should be revisited using more powerful technologies now available.

The idea of environmentally-induced transposition has also arisen from analysis of the growing number of available complete prokaryote genome sequences. These have identified many bacterial and archaeal species in which the number of ISs is dramatically high. They include different *Shigellae* species [33], *Bordetella pertusis* [34], *Yersinia pestis* [35]–[36] *Lactobacillus* [37], *Sulfolobus solfataricus* [38] among many others. While it is attractive to imagine that this is the result of transposition bursts induced by an environmental trigger, a strong alternative has been to invoke stochastic transposition together with the formation of population bottlenecks produced in small isolated populations to fix such mutational events (insertions) [34].

Irradiation-triggered transposition described here is a response to an extreme set of environmental conditions which transiently generates large quantities of a substrate (single strand DNA) favoring transposition of IS*Dra2* but not of the other, unrelated, ISs present in the *D. radiodurans* genome. This response would result in movement of the single “top strand” IS copy generating mutational diversity while retaining the inactive bottom strand copy. Insertion of the single-stranded circle intermediate and replication of the bottom strand would increase the copy number of the IS and disperse it throughout the genome. Moreover, *D. radiodurans* possesses an efficient natural competence system whose regulation remains to be explored. In view of the fact that such processes in other bacterial species occur using single strand DNA intermediates, transformation may be instrumental in assuring spread of this single strand transposable element [39]. Thus radiation-triggered IS*Dra2* transposition might additionally generate diversity through participation of DNA containing newly transposed IS*Dra2* copies in intercellular transformation.

An increase in the IS*Dra2* copy number exposed to a single gamma irradiation cycle has been observed previously (see Figure S2 in [19]). Moreover, Islam et al [20] characterized the distribution of IS*Dra2* in different laboratory *D. radiodurans* strains and found that its copy number varied from strain to strain: 7 copies were identified in the published *D. radiodurans* R1 genome sequence, 6 in the MR1 strain, 21 in the KD8301 strain but only one in KR1, the parent strain of KD8301 and in the reference strain ATCC 13939 used here. Unfortunately, the history and the intermediate strains are not available and no conclusions can be drawn concerning the factors which contributed to the gain or loss of IS*Dra2* copies in the genome of these strains. The expansion of transposon copy number with accompanying ectopic sequence homology raises the question whether this would be a detriment to the extensive chromosome repair process after heavy irradiation. Precise reconstitution of a shattered genome through ESDSA involves annealing of 20–30 kb single-stranded DNA overhangs. Zahradka et al ([18]) speculated that short blocks, 1–2 kb, of dispersed repetitive sequences present in the genome of *D. radiodurans* would not compromise the accuracy of repair through ESDSA. These authors pointed out that annealing only a limited repeated sequence block within two long non-complementary single-stranded overhangs could not readily link two fragments together. However, such ectopic sequence homology might stimulate chromosomal rearrangements through other DNA double strand break repair pathway such as single strand annealing (SSA) with a potential of increasing the plasticity of the genome under very adverse conditions.

Since this class of newly recognized transposable elements require single stranded DNA as both substrate and target site, any process which generates single strand DNA such as replication, mismatch repair and transcription, might lead similarly to limited induction of transposition and also provide suitable insertion sites. In view of the extremely widespread occurrence of members of this IS family in nature, this type of mechanism could be important in regulating transposition activity, interfacing transposition with host physiology and cell cycle and in creating genomic diversity as has been recently shown for the IS*200*/*605* family member, IS*1541*, whose insertion allows the *Yersinia pestis* host to escape from adaptive immune responses and plague immunity [9].

## Materials and Methods

### Bacterial strains, media, and growth conditions

Bacterial strains are listed in Table S1. *E. coli* strain DH5α was the general cloning host and strain SCS110 was used to propagate plasmids prior to introduction into *D. radiodurans* via transformation[40]. All *D. radiodurans* strains were derivatives of strain R1 (ATCC 13939). TGY2X liquid medium and TGY plates [41] were used for *D. radiodurans* and Luria-Bertani (LB) broth for *E. coli* strains. Media were supplemented with the appropriate antibiotics used at final concentrations of: chloramphenicol 20 µg ml^−1^ for *E. coli* and 3 µg ml^−1^ for *D. radiodurans*; spectinomycin 40 µg ml^−1^ for *E. coli* and 75 µg ml^−1^ for *D. radiodurans*; tetracycline 2.5 µg ml^−1^ for *D. radiodurans*; hygromycin 50 µg ml^−1^. 5% (w/v) sucrose was supplemented to isolate the *D. radiodurans sacB* inactivated mutants.

Transformation of *D. radiodurans* with genomic DNA, PCR products, or plasmid DNA was performed as described [41].

### DNA manipulations

Plasmid DNA was extracted from *E. coli* using the QIAprep Spin miniprep kit (Qiagen). *D. radiodurans* chromosomal DNA was isolated as described previously [19]. PCR reactions were carried out with Phusion DNA Polymerase (Abgene).

Inverse PCR was performed as follows: genomic DNA was digested with *Nar*I, purified by Phase Lock Gel procedure (Eppendorf), and then ligated using T4 DNA ligase. After ethanol precipitation, the ligated circular DNA was used as a template with primers P3 and P4 described in Table S1. The iPCR products were then directly sequenced with LEext and REext by Genome Express (Grenoble, France). Oligonucleotides used are listed in Table S3.

PCR reactions used for analysis of kinetics of irradiation-induced IS*Dra2* excision were performed as follows: PCR was carried out in a final volume of 50 µl with using 0.5 Units of DyNazyme EXT DNA polymerase (Finnzymes) and 200 µM of each dNTP. To detect the donor joint, PCR analysis using P1+P2 primer pair was performed under the following conditions: 94°C for 3 min, 30 cycles of 94°C for 45 s, 60°C for 45 s, and 72°C for 20 s; and finally 72°C for 10 min. To detect the RE-LE junction, PCR analysis using P3+P4 was carried out as follows: the PCR of the first round was performed with 1 µg of genomic DNA under the following conditions: 94°C for 3 min, 30 cycles of 94°C for 45 s, 55°C for 45 s, and 72°C for 10 s, and finally 72°C for 10 min. The second round of PCR used a 15 µl aliquot from round 1 as template and was done under the same conditions than round 1.

### Plasmids

For plasmid pGY13224 expressing *tnpA* and *tnpB* from a P*_spac_* promoter, the coding sequences of the two genes were amplified by PCR using primers DraF (tagged with *Eco*RV) and DraR (tagged with *Xho*I). After cleavage, the PCR fragment was cloned into pGY11559 between the *Swa*I and *Xho*I sites.

For plasmid pGY13203 expressing *tnpA* from the P*_spac_* promoter, the *D. radiodurans* IS*Dra2 tnpA* gene was amplified by PCR using the primer pair DraF/DraX and *D. radiodurans* R1 genomic DNA as template. The product was cloned into plasmid pGY11559 between the *EcoR*V and *Xho*I sites.

For plasmid pGY13204 expressing *tnpB* from the P*_spac_* promoter, the N-terminal part of *tnpB* was amplified using the primer pair 1651F/1651R, digested with *Nde*I and *Bsa*I and ligated to the 10885-bp *Nde*I-*Bsa*I fragment from pGY13224.

For plasmid pGY13507 expressing *sacB* from the P*_spac_* promoter, the *B. subtilis sacB* gene was amplified with the primer pair NdeUPsacB/XhoDwnsacB and cloned into pGY11559 between the *Nde*I and *Xho*I sites.

For plasmid pGY11556 expressing the *E. coli lacZ* from the P*_spac_* promoter, pGY11559 was digested with *Bgl*II and *Xho*I and ligated to the *Bgl*II-*Xho*I fragment from pGY11540 containing *lacZ* fused to the P*_spac_* promoter.

#### Plasmids for production and *in vivo* analysis of His-tagged TnpA

The *tnpA* coding sequence was amplified with the primer pair NdeUptnpA/TnpAHISsph and was cloned at the *Nde*I and *Sph*I sites of pAPT110 with a C-terminal His_6_ tag under the control of P*_lac_*, to generate plasmid pGY13503. The plasmid also carried the *lacI* gene to regulate *tnpA*-His_6_ expression. To analyse the ability of C-terminal His-tagged TnpA to catalyse *in vivo* transposition, the *tnpA*-His_6_ copy was amplified from pGY13503 with primer UpNde107 (tagged with *Nde*I) and DwntnpAHISxho (tagged with *Xho*I) and cloned into pGY11559 at the unique *Nde*I and *Xho*I sites to produce plasmid pGY13505, in which *tnpA*-His_6_ is expressed under the control of P*_spac_* promoter.

### Pulsed field gel electrophoresis

Irradiated cultures were diluted in TGY2X to an A_650_ = 0.3 and incubated at 30°C. At different post-irradiation incubation times, samples (5 ml) were taken to prepare DNA plugs as described [42]. The DNA in the plugs was digested for 16 h at 37°C with 60 units of *Not*I restriction enzyme. After digestion, the plugs were subjected to pulsed field gel electrophoresis for 28 hours at 10°C using a CHEF MAPPER electrophoresis system (Biorad) with the following conditions: 5.5 V/cm, linear pulse of 40 s, and a switching angle of 120° (−60° to +60°).

### Measurement of *in vivo* spontaneous, γ-, and UV-induced excision frequencies of IS*Dra2* derivatives

Individual Cam^R^ Tet^S^ colonies purified from GY13111 or derivatives of GY13115 strain expressing *in trans* TnpA, or TnpB or no protein were inoculated into 3 ml of TGY2X supplemented with spectinomycin when required and grown to an A_650_ of 1–2. The bacterial cultures were washed in 10 mM MgSO_4_, resuspended in the same buffer to an A_650_ = 1. Half of the resuspension was kept on ice, and the second half was exposed to UV light at a dose rate of 3.5 J m^−2^ s^−1^ in Petri dishes. For γ-irradiation, the cultures were grown to an A_650_ = 1, then concentrated 30-fold in TGY2X and irradiated on ice with a ^137^Cs irradiation system (Institut Curie, Orsay, France) at a dose rate of 41.8 Gy min^−1^.

UV-, γ- or non-irradiated cells were diluted in TGY2X to an A_650_ = 0.3 and grown to stationary phase. Determination of the total number of viable cells was performed on TGY plates and excision of IS*Dra2* derivatives from *tetA* gene was selected on TGY plates containing tetracycline. Colonies were counted after 3–4 days of incubation at 30°C. The frequencies of the excision event per viable cell from 10 independent experiments were used to calculate the mean values and the standard deviations.

### Measurement of *in vivo* spontaneous and γ-induced insertion frequencies of IS*Dra2*-103 into *sacB*

5 individual Cam^R^ Tet^S^ colonies purified from GY13174 strain expressing IS*Dra2*-103 were inoculated into 10 ml of TGY2X supplemented with hygromycin and grown to an A_650_ of 1.5. The bacterial cultures were concentrated 30-fold in TGY2X. 100 µl of the resuspension was kept on ice and the rest was γ-irradiated as described above. After dilution, γ- or non-irradiated cells were grown to stationary phase. Excision of IS*Dra2*-103 from *tetA* and insertion into *sacB* gene were selected on TGY plates containing tetracycline, or tetracycline, chloramphenicol and sucrose, respectively. Colonies were counted after 3–4 days of incubation at 30°C. The insertion frequencies per viable cell from 5 independent experiments were used to calculate the mean values and the standard deviations.

### Measurement of *lacZ* expression under control of P*_tnpA_* or P*_spac_* promoters

Replacement of the *tnpA* coding region of IS*Dra2*-103 with the *lacZ* coding region to generate IS*Dra2*-104 (Figure 1A) was performed as follows: the P*_tnpA_*::*lacZ* fusion was amplified by the joining PCR method [43]; see Table S3). The resulting *lacZ* fusion, the accompanying chloramphenicol resistance cassette and the right end of IS*Dra2*-103 were then inserted into the *tetA* gene of strain GY13109 using the tripartite ligation method [44]. The resulting strain GY14310 was selected for Cam^R^ and the insertion of the *lacZ* fusion by homologous recombination was confirmed by diagnostic PCR. *LacZ* gene was expressed under the P*_spac_* promoter in strain GY14312 containing plasmid pGY11556. Expression of the *lacZ* reporter gene was detected in *D. radioduran*s colonies formed on TGY plates containing 5-bromo-4-chloro-3-indolyl-*β*-D-galactoside (X-gal) at 40 µg/ml. β-galactosidase activity was measured as previously described [41].

### TnpA purification, DNA procedures, and oligonucleotide cleavage and strand transfer reactions in vitro

TnpA was purified from *E. coli* K12 MC1061 *endA* carrying TnpA-His_6_ expression plasmid pGY13503 (Table S1) following induction with 0.5 mM IPTG as previously described [25].

#### Oligonucleotide substrates for *in vitro* reactions

LE (59-mer):

GGCGTCTGAATGGCCTTGATGCTTGAGGGGCGCACACTCGTGACTTCAGTCATGAGTTA

LEcom (59-mer):

TAACTCATGACTGAAGTCACGAGTGTGCGCCCCTCAAGCATCAAGGCCATTCAGACGCC

RE (63-mer):

CTGCGAAGTGAGAATCACGCGACTTTAGTCGTGTGAGGTTCAAGAGTCCCTTGGCGCCCATGA

REcom (63-mer)

TCATGGGCGCCAAGGGACTCTTGAACCTCACACGACTAAAGTCGCGTGATTCTCACTTCGCAG

### *In vitro* oligonucleotide cleavage and strand transfer reactions

Reactions were performed by 45 min incubation of 20 fmol of a 5′-^32^P-labelled oligonucleotide and 1 pmol of the same oligonucleotide unlabelled with or without 10 pmol unlabelled recombining oligonucleotide, 0.5 µg of poly-dIdC and 20 pmol TnpA-His_6_ at 37°C in a final volume of 16 µl in 20 mM HEPES (pH 7.5), 2.5% DMSO, 200 mM NaCl, 5 mM MgCl_2_, 1 mM TCEP, 20 µg/ml BSA and 10% glycerol. The reactions were terminated by addition of 0.1% SDS followed by 15 min of incubation at 37°C and separated on a 10% denaturing sequencing polyacrylamide gel. The gel was analysed by phosphorimaging.

#### 5′-end-labelling

10 pmol of oligonucleotide was mixed with 16 pmol of [γ-^32^P] ATP (5000 Ci/mmol, Amersham Inc.) and 1 unit of T4 kinase (NEB Inc.) in T4 kinase buffer (70 mM Tris–HCl pH 7.6, 10 mM MgCl_2_, 5 mM DTT). Incubation was for 1 h at 37°C. Labelled oligonucleotides were purified by filtration through Sephadex G25. Ds substrates were obtained by hybridisation of labelled top-stranded oligonucleotide with cold bottom-stranded oligonucleotide.

## Supporting Information

Figure S1 Construction of the *D. radiodurans* tester strains. All constructions described below were verified by DNA sequencing. The oligonucleotides used for PCR amplification of DNA fragments required for strains or plasmids construction, for diagnostic PCR or for sequencing are described in Table S3. The active IS*Dra2* genomic copy (loci *DR1651-DR1652*) was first replaced with a Tet^R^ cassette expressing the *tetA* gene from the deinococcal P*_groESL_* promoter using the tripartite ligation method [Mennecier S, Coste G, Servant P, Bailone A, Sommer S: 2004 *Mol Genet Genomics*, **272**(4):460–469.]. The resulting strain, GY13109, was selected for its Tet^R^ phenotype and the allelic replacement of IS*Dra2* by the Tet^R^ cassette was confirmed by diagnostic PCR. The IS*Dra2* derivatives (Figure 1A) were inserted at the unique 5′TTGAT3′ target sequence of the *tetA* gene by double-crossover events between tripartite ligation products previously amplified by the joining PCR method [Fabret C, Ehrlich SD, Noirot P: 2002 *Mol Microbiol*, **46**(1):25–36.] and chromosomal *tetA* region. The resulting tester strains (GY13115 with IS*Dra2*-113; GY13111 with IS*Dra2*-103; and GY13173 with IS*Dra2*-103Term116; Figure 1B) were selected for Cam^R^ and the insertion of the IS*Dra2* derivatives into the *tetA* gene by homologous recombination was confirmed by diagnostic PCR. Transposon insertion into *sacB*, was studied by first replacing the degenerate ISDra2* copy (loci *DR0177*-*DR0178*) with the *sacB* gene from *B. subtilis* and the accompanying hygromycin resistance cassette. The strain GY13173 or GY13177 was transformed by the tripartite ligation mixture (see Table S2) and the resulting strains GY13174 and GY13182, respectively, were selected for their Hyg^R^ phenotype. The allelic replacement of ISDra2* by the fragment encompassing the Hyg^R^ cassette and the *sacB* gene was confirmed by diagnostic PCR and sequenced.
(0.46 MB TIF)

Figure S2Expression of the *lacZ* reporter gene under the control of P*_tnpA_* or P*_spac_* promoter. GY14310 and GY14312 bacteria expressing *lacZ* under the control of P*_tnpA_* and P*_spac_* respectively were exposed (▪) or not (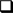) to 5 kGy γ-irradiation and β-galactosidase activity was measured at time 0, 30, 60, 120, and 180 min post irradiation incubation. The strain GY14312 was grown in the absence (-IPTG) or in the presence of 1 mM IPTG (+IPTG). The β-galactosidase activity being constant at the different times after irradiation, the results were presented only for t = 60 min. Values are averages±standard deviation derived from three independent experiments.(0.48 MB TIF)

Figure S3 PCR analyses of Suc^R^ insertion mutants. (A) Schematic representation of the *sacB* gene showing potential pentanucleotide insertion sites (S1 - 10) and the position of primers used for PCR analysis. (B) Agarose gel showing the sizes of amplification products obtained with genomic DNA of 10 Tet^R^ Cam^R^ Suc^R^ mutants (from strain GY13186) as template and the pair of primers Camdwn/RevsacB. Mutants sac1, 3, 4, and 7 are inserted at site S1 of *sacB*; mutant sac2 is inserted at site S8; mutants sac8 and sac9 at site S9 and mutants sac5, sac6, and sac10 are inserted at site S5.
(1.67 MB TIF)

Table S1 Bacterial strains and plasmids.
(0.06 MB DOC)

Table S2 *ISDra2-113* genomic insertions sites.
(0.03 MB DOC)

Table S3 Overview of primers used for strains construction, cloning, diagnostic PCR, and sequencing experiments.
(0.16 MB DOC)
