# Supplementary figures and images for: Irradiation-Induced Deinococcus radiodurans Genome Fragmentation Triggers Transposition of a Single Resident Insertion Sequence
Source: PLoS Genet. 2010 Jan 15;6(1):e1000799. doi: 10.1371/journal.pgen.1000799 (PMC2806898; doi:10.1371/journal.pgen.1000799)

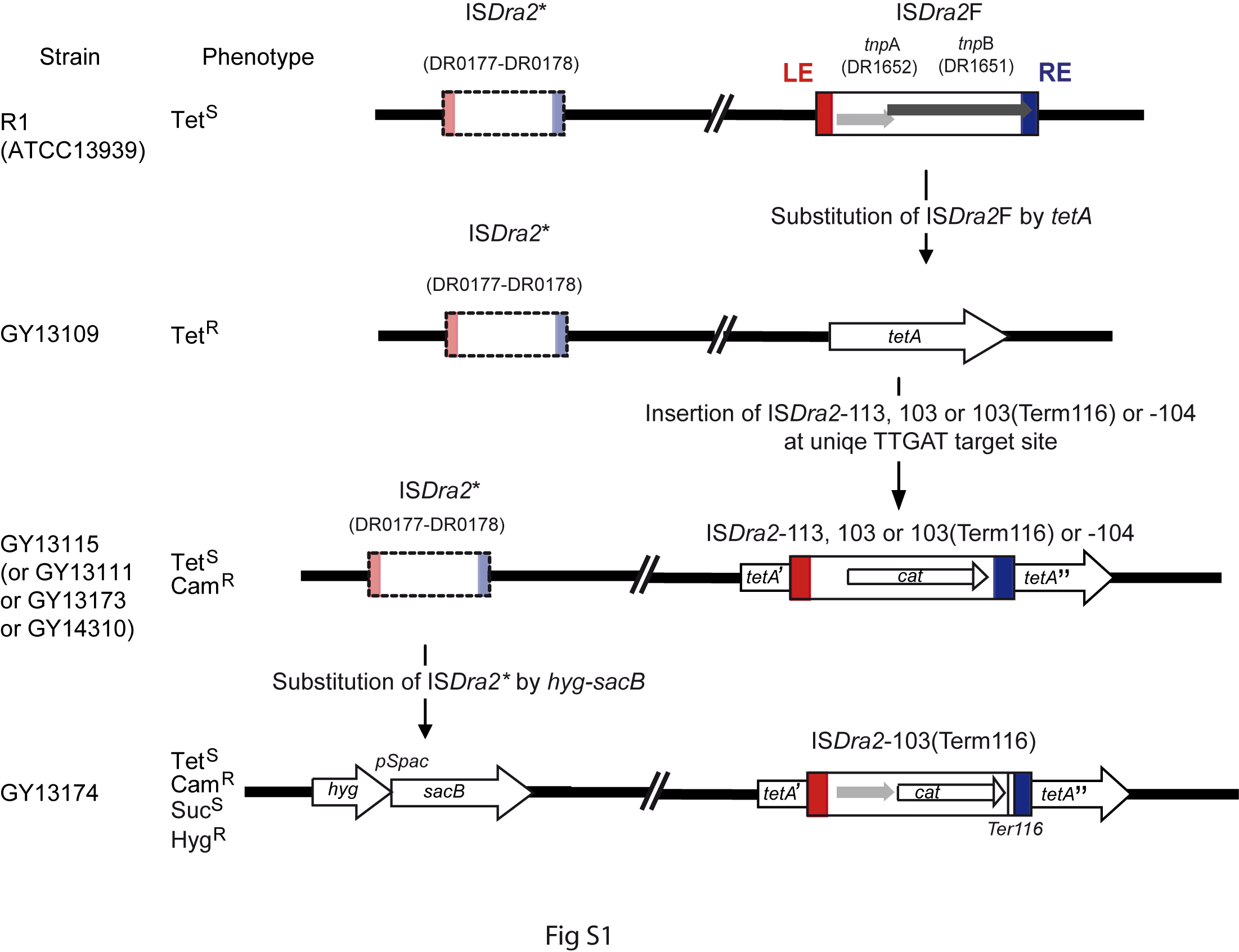

Supplement: Figure S1 — Construction of the D. radiodurans tester strains. All constructions described below were verified by DNA sequencing. The oligonucleotides used for PCR amplification of DNA fragments required for strains or plasmids construction, for diagnostic PCR or for sequencing are described in Table S3. The active ISDra2 genomic copy (loci DR1651-DR1652) was first replaced with a TetR cassette expressing the tetA gene from the deinococcal PgroESL promoter using the tripartite ligation method [Mennecier S, Coste G, Servant P, Bailone A, Sommer S: 2004 Mol Genet Genomics, 272(4):460–469.]. The resulting strain, GY13109, was selected for its TetR phenotype and the allelic replacement of ISDra2 by the TetR cassette was confirmed by diagnostic PCR. The ISDra2 derivatives (Figure 1A) were inserted at the unique 5′TTGAT3′ target sequence of the tetA gene by double-crossover events between tripartite ligation products previously amplified by the joining PCR method [Fabret C, Ehrlich SD, Noirot P: 2002 Mol Microbiol, 46(1):25–36.] and chromosomal tetA region. The resulting tester strains (GY13115 with ISDra2-113; GY13111 with ISDra2-103; and GY13173 with ISDra2-103Term116; Figure 1B) were selected for CamR and the insertion of the ISDra2 derivatives into the tetA gene by homologous recombination was confirmed by diagnostic PCR. Transposon insertion into sacB, was studied by first replacing the degenerate ISDra2* copy (loci DR0177-DR0178) with the sacB gene from B. subtilis and the accompanying hygromycin resistance cassette. The strain GY13173 or GY13177 was transformed by the tripartite ligation mixture (see Table S2) and the resulting strains GY13174 and GY13182, respectively, were selected for their HygR phenotype. The allelic replacement of ISDra2* by the fragment encompassing the HygR cassette and the sacB gene was confirmed by diagnostic PCR and sequenced. (0.46 MB TIF) [file pgen.1000799.s001.tif]

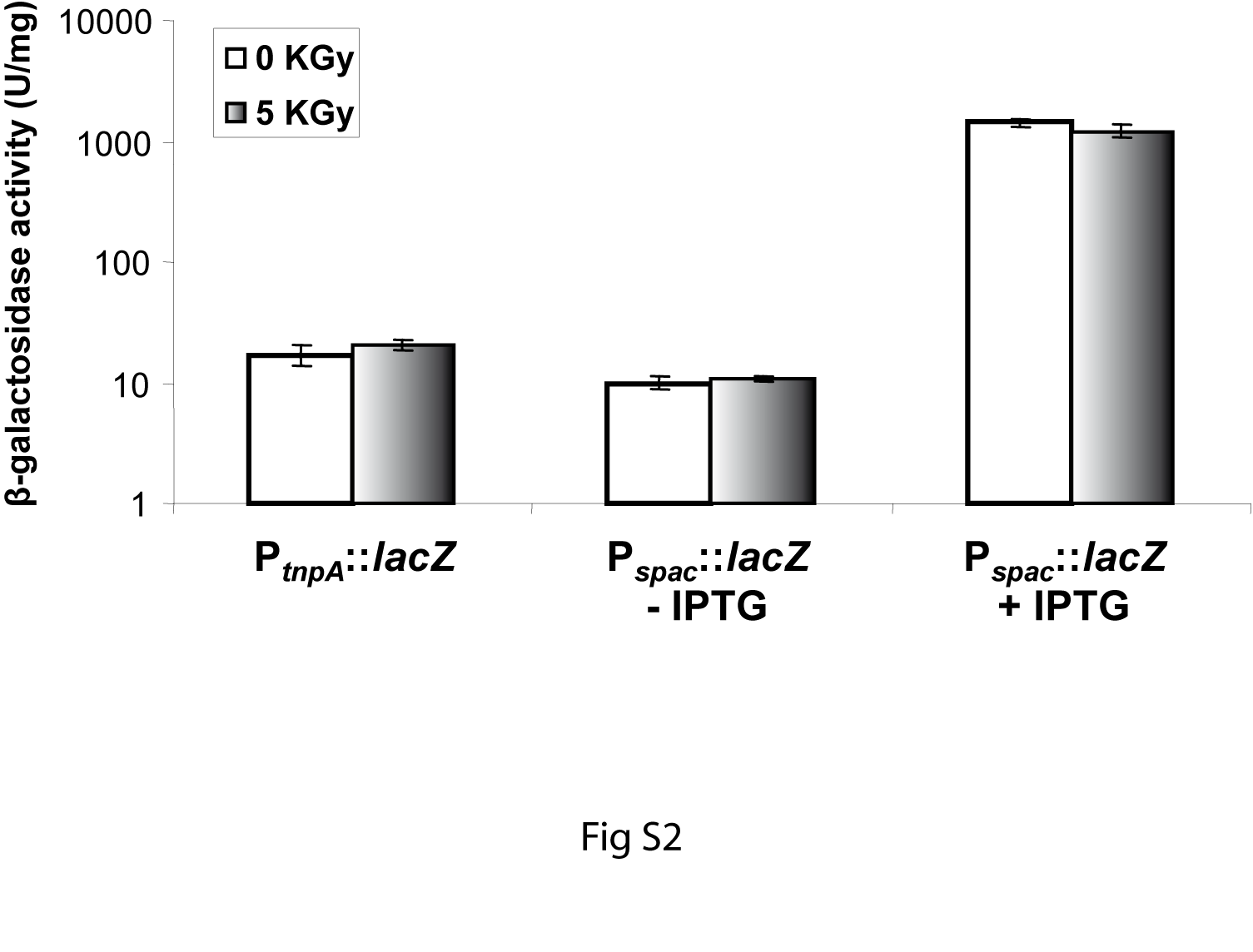

Supplement: Figure S2 — Expression of the lacZ reporter gene under the control of PtnpA or Pspac promoter. GY14310 and GY14312 bacteria expressing lacZ under the control of PtnpA and Pspac respectively were exposed (▪) or not () to 5 kGy γ-irradiation and β-galactosidase activity was measured at time 0, 30, 60, 120, and 180 min post irradiation incubation. The strain GY14312 was grown in the absence (-IPTG) or in the presence of 1 mM IPTG (+IPTG). The β-galactosidase activity being constant at the different times after irradiation, the results were presented only for t = 60 min. Values are averages±standard deviation derived from three independent experiments. (0.48 MB TIF) [file pgen.1000799.s002.tif]

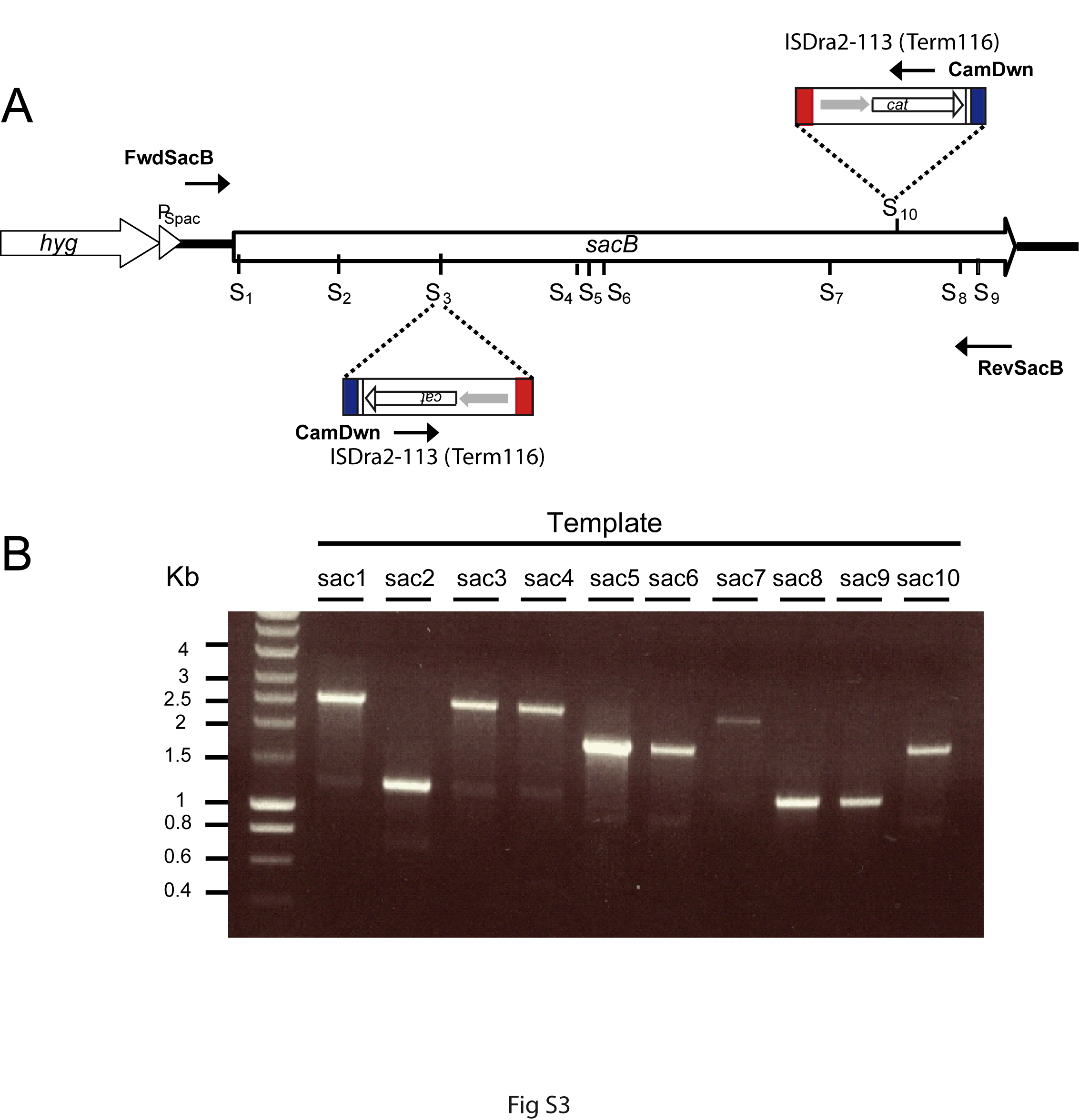

Supplement: Figure S3 — PCR analyses of SucR insertion mutants. (A) Schematic representation of the sacB gene showing potential pentanucleotide insertion sites (S1 - 10) and the position of primers used for PCR analysis. (B) Agarose gel showing the sizes of amplification products obtained with genomic DNA of 10 TetR CamR SucR mutants (from strain GY13186) as template and the pair of primers Camdwn/RevsacB. Mutants sac1, 3, 4, and 7 are inserted at site S1 of sacB; mutant sac2 is inserted at site S8; mutants sac8 and sac9 at site S9 and mutants sac5, sac6, and sac10 are inserted at site S5. (1.67 MB TIF) [file pgen.1000799.s003.tif]
