# Supplementary material for: Irradiation-Induced Deinococcus radiodurans Genome Fragmentation Triggers Transposition of a Single Resident Insertion Sequence
Source: PLoS Genet. 2010 Jan 15;6(1):e1000799. doi: 10.1371/journal.pgen.1000799 (PMC2806898; doi:10.1371/journal.pgen.1000799)
Supplement: Table S1 — Bacterial strains and plasmids. (0.06 MB DOC) [file pgen.1000799.s004.doc]

**Table S1.** Bacterial strains and plasmids

| Strain or plasmid | Genotype or other relevant characteristics | Source or reference |
| --- | --- | --- |
| *E. coli* | | |
| DH5α | *supE44 hsdR17 recA1 endA1 lacZ*Δ*M15* | Laboratory stock |
| SCS110 | *endA dam dcm supE44* Δ(*lac-proAB*) (*F’traD36 proAB lacI^q^Z*Δ*M15*) | Laboratory stock |
| *D. radiodurans* | | |
| R1 | ATCC 13939 | Anderson AW, et al. 1956 *Food Technol*, 10:575-578. |
| GY13109 | As R1 but Δ(IS*Dra2*F)Ω*tetA* | This work |
| GY13111 | GY13109 *tetA*ΩIS*Dra2*-103 | This work |
| GY13115 | GY13109 *tetA*ΩIS*Dra2*-113 | This work |
| GY13119 | GY13115 (pGY11559) | This work |
| GY13120 | GY13115 (pGY13203) | This work |
| GY13121 | GY13115 (pGY13204) | This work |
| GY13173 | GY13109 *tetA*ΩIS*Dra2*-103Term116 | This work |
| GY13177 | GY13109 *tetA*ΩIS*Dra2*-113Term116 | This work |
| GY13174 | GY13173 Δ(IS*Dra2**)Ω(P*_kat_*::*hph* *sacB*) | This work |
| GY13182 | GY13177 Δ(IS*Dra2**)Ω(P*_kat_*::*hph* *sacB*) | This work |
| GY13186 | GY13182 (pGY13203) | This work |
| GY14310 | GY13109 *tetA*ΩIS*Dra2*-104 | This work |
| GY14312 | GY13115 (pGY11556) | This work |
|  |  |  |
| **Plasmids** |  |  |
| pAPT110 | Expression vector in *E. coli* for protein purification | Polard P, Chandler M 1995 *Genes Dev*, **9**(22):2846-2858. |
| pGTC101 | Source of a P*_tufA_*::*cat* cassette in *D. radiodurans* | Earl AM, et al., 2002 *J Bacteriol*, **184**(4):1003-1009. |
| pZT29 | Source of a P*_kat_*::*cat* cassette in *D. radiodurans* | Satoh K, et al., 2009 *Plasmid*, **62** (1):1-9. |
| pGY12724 | Source of a Cam^R^ Term116 cassette in *D. radiodurans* | Laboratory stock |
| pGY11615 | Source of a Tet^R^ cassette in *D. radiodurans* | Mennecier S, et al., 2004 *Mol Genet Genomics*, **272**(4):460-469. |
| pKatHPH4 | Source of a Hyg^R^ cassette in *D. radiodurans* | Gift of I. Narumi |
| pGY11559 | Expression vector; P*_spac_*,P*_tufA_*::*lacI*, Spc^R^ in *E.coli* and *D.radiodurans* | Mennecier S, et al., 2004 *Mol Genet Genomics*, **272**(4):460-469. |
| pGY13203 | pGY11559; P*_spac_*::*tnpA* | This work |
| pGY13204 | pGY11559; P*_spac_*::*tnpB* | This work |
| pGY13224 | pGY11559; P*_spac_*::*tnpAtnpB* | This work |
| pGY13503 | pAPT110; P*_lac_*::*tnpA*His_6_ | This work |
| pGY13505 | pGY11559; P*_spac_*::*tnpA*His_6_ | This work |
| pGY13507 | pGY11559; P*_spac_*::*sacB* | This work |
| pGY11540 | Source of P*_spac_*::*lacZ* | Lecointe F, et al. 200*4 Ge*ne**, 33**6 (1):25-35.7] |
| pGY11556 | pGY11559; P*_spac_*::*lacZ* | This work |
