# Supplementary material for: Irradiation-Induced Deinococcus radiodurans Genome Fragmentation Triggers Transposition of a Single Resident Insertion Sequence
Source: PLoS Genet. 2010 Jan 15;6(1):e1000799. doi: 10.1371/journal.pgen.1000799 (PMC2806898; doi:10.1371/journal.pgen.1000799)
Supplement: Table S2 — ISDra2-113 genomic insertions sites. (0.03 MB DOC) [file pgen.1000799.s005.doc]

**Table S2:** *ISDra2-113* genomic insertions sites.

| Site of insertion  (genomic coordinate^(a)^) | Disrupted gene | Number  of occurrences ^(b)^ |
| --- | --- | --- |
| 1336081 (I) | DR1331 | 1 |
| 1336156 (I) | DR1331 | 1 |
| 1337411 (I) | DR1333 | 1 |
| 1844571 (I) | DR1819 | 1 |
| 2089228 (I) | DR2073 | 1 |
| 1679887 (I) | DR1655 | 3 |
| 1679155 (I) | DR1655 | 1 |
| 1497908 (I) | DR1483 | 1 |
| 10827 (I) | DR0010 | 1 |
| 1221633 (I) | DR1213 | 1 |
| 1673725 (I) | DR1649 | 2 |
| 373802 (II) | DRA0341 | 1 |
| 1197081 (I) | DR1188 | 1 |

^(a)^ Refers to the first nucleotide 3’ to the target sequence 5’-TTGAT-3’. Romanic numbers in brackets refer to chromosomes.

^(b)^ On a sample of 16 independent spontaneous Tet^R^ Cam^R^ isolates of the tester strain GY13120 expressing TnpA protein in *trans*.
