# Supplementary material for: Irradiation-Induced Deinococcus radiodurans Genome Fragmentation Triggers Transposition of a Single Resident Insertion Sequence
Source: PLoS Genet. 2010 Jan 15;6(1):e1000799. doi: 10.1371/journal.pgen.1000799 (PMC2806898; doi:10.1371/journal.pgen.1000799)
Supplement: Table S3 — Overview of primers used for strains construction, cloning, diagnostic PCR, and sequencing experiments. (0.16 MB DOC) [file pgen.1000799.s006.doc]

**TABLE S3**. Overview of primers used for strains construction, cloning, diagnostic PCR and sequencing experiments.

| **Primer** | **Primers sequences (5' ⭢ 3')^a^** | ***Use*** |
| --- | --- | --- |
|  | | |
| **Replacement of IS*Dra2*F by *tetA* (tripartite ligation procedure, strain GY13109)** | | |
| 50XHO | CAGA**CTCGAG**CCATGAAGGCGAAGAGGATA | Amplification of a DR1650 portion downstream of IS*Dra2* |
| TESTB | AATGGTCGAAGCCGCCGAGA (c) |
| K7TETPST | TGTTGCCATTG**CTGCAG**GAG | Amplification of a *tet^R^* cassette from pGY11615 |
| K7TETXHO | CATA**CTCGAG**CGGCTTCCATTCAGGTCGAG (c) |
| 53PST | CAGA**CTGCAG**CTACACCTGGCCGCAACTGA | Amplification of a DR1653 portion upstream of IS*Dra2* |
| A2 | GTTCTGCGCCATGATGAAGG (c) |
|  | | |
| **Construction of IS*Dra2*-103 derivative (strain GY13111)** | | |
|  | | |
| **Amplification of L-Jun103 fragments** | | |
| K7TETPST | TGTTGCCATTG**CTGCAG**GAG | Amplification of the L-JunA fragment containing the *tetA* portion and the target site |
| TAIL-LE | CGCCCCTCAAGCATCAACGCATATAGCGCTAG (c) |
| TAIL-TET | CTATATGCGTTGATGCTTGAGGGGCGCACACT | Amplification of the L-JunB103 fragment containing the left end of IS*Dra2* including *tnpA* coding sequence |
| NEWC1 | GAAC**GGATCC**GAAAGCCTTATTCCTTATCA (c) |
|  | | |
| **Amplification of R-Jun fragments** | | |
| RF | CCATGTCGGCAGAATGCTTA | Amplification of R-JunA fragment containing the RE of IS*Dra2* |
| RTAIL-TET | GCATAGAAATTGCTTGAACCTCACACGACTAA (c) |
| RTAIL-RE | GTGTGAGGTTCAAGCAATTTCTATGCGCACC | Amplification of R-JunB fragment containing the *tetA* portion downstream of the target site |
| RR | CTCTCCCTTATGCGACTCCT (c) |
|  | | |
| **Amplification of fusion fragments (joining PCR procedure)** | | |
| K7TETPST | TGTTGCCATTG**CTGCAG**GAG | Amplification of the L-Jun103 fusion fragment from a mixture of overlapping L-JunA and L-JunB103 fragments |
| NEWC1 | GAAC**GGATCC**GAAAGCCTTATTCCTTATCA (c) |
| RF | CCATGTCGGCAGAATGCTTA | Amplification of the R-Jun fusion fragment from a mixture of overlapping R-JunA and R-JunB fragments |
| RR | CTCTCCCTTATGCGACTCCT (c) |
|  | | |
| **Construction of IS*Dra2*-113 derivative (strain GY13115)** | | |
|  | | |
| **Amplification of L-Jun113 fragments** | | |
| K7TETPST | TGTTGCCATTG**CTGCAG**GAG | Amplification of the L-JunA fragment containing the *tetA* portion and the target site |
| TAIL-LE | CGCCCCTCAAGCATCAACGCATATAGCGCTAG (c) |
| TAIL-TET | CTATATGCGTTGATGCTTGAGGGGCGCACACT | Amplification of the L-JunB113 fragment containing the LE of IS*Dra2* |
| LE-113 | TTGGATCCTTCTGAGCGGCAATGTCACG (c) |
|  | | |
| **Amplification of R-Jun fragments** | | |
| RF | CCATGTCGGCAGAATGCTTA | Amplification of R-JunA fragment containing the RE of IS*Dra2* |
| RTAIL-TET | GCATAGAAATTGCTTGAACCTCACACGACTAA (c) |
| RTAIL-RE | GTGTGAGGTTCAAGCAATTTCTATGCGCACC | Amplification of R-JunB fragment containing the *tetA* portion downstream of the target site |
| RR | CTCTCCCTTATGCGACTCCT (c) |
|  | | |
| **Amplification of fusion fragments (joining PCR procedure)** | | |
| K7TETPST | TGTTGCCATTG**CTGCAG**GAG | Amplification of the L-Jun113 fusion fragment from a mixture of overlapping L-JunA and L-JunB113 fragments |
| LE113 | TTGGATCCTTCTGAGCGGCAATGTCACG (c) |
| RF | CCATGTCGGCAGAATGCTTA | Amplification of the R-Jun fusion fragment from a mixture of overlapping R-JunA and R-JunB fragments |
| RR | CTCTCCCTTATGCGACTCCT (c) |
|  | | |
| **Amplification of the Cam^R^ cassette** | | |
| CAM1 | GGCC**GGATCC**ACGGAACCTATACGGGAACT | Amplification of the Cam^R^ cassette from plasmid pGTC101 |
| CAM2 | GGTC**TCTAGA**GCACTTATTCAGGCGTAGCA (c) |
|  | | |
| **Construction of IS*Dra2*-103Term116 derivative (tripartite ligation procedure, strain GY13173)** | | |
| D52F | CTGCAGGAGCGTCATAGACT | Amplification of a fragment encompassing the junction *tetA*-LE and the DR1652 orf from genomic DNA of GY13111 |
| PS93 | TATCCAGCTGAACGGTCTGGTTA (c) |
| UpCmBam | TGT**GGATCC**CGATGACAAGTAGTGATA | Amplification of the Cam^R^Term116 cassette from plasmid p12724 |
| DT116Xba | CTCTG**TCTAGA**ACGCGTTGGGAGCTCTCC (c) |
| RF | CCATGTCGGCAGAATGCTTA | Amplification of the R-Jun fragment from genomic DNA of GY13115 |
| RR | CTCTCCCTTATGCGACTCCT (c) |
|  | | |
| **Construction of IS*Dra2*-113Term116 derivative (tripartite ligation procedure, strain GY13177)** | | |
| D52F | CTGCAGGAGCGTCATAGACT | Amplification of a fragment encompassing the junction *tetA*-LE from genomic DNA of GY13115 |
| D52RBam | GCAT**GGATCC**TTCTGAGCGGCAATGTCACG (c) |
| UpCmBam | TGT**GGATCC**CGATGACAAGTAGTGATA | Amplification of the Cam^R^Term116 cassette from plasmid p12724 |
| DT116Xba | CTCTG**TCTAGA**ACGCGTTGGGAGCTCTCC (c) |
| RF | CCATGTCGGCAGAATGCTTA | Amplification of the R-Jun fragment from genomic DNA of GY13115 |
| RR | CTCTCCCTTATGCGACTCCT (c) |
|  | | |
| **Replacement of IS*Dra2** by hyg^R^-P*_spac_::sacB* (strains GY13174 and GY13182)** | | |
| 176UP | TTGCAGCGCCTGGGCCTGGACC | Amplification of the DR0176 portion |
| 176BglII | GGCC**AGATCT**TGCAGCGCCCGCAGCGTC (c) |
| 179Xho | GCGC**CTCGAG**CTACACTGTCGGGCGAGATGC | Amplification of the DR0179 portion |
| 179Dwn | GTGGCTGATGGCCTGCTTCTG (c) |
| FwdBglII | GGCC**AGATCT**ATCGAATTCGAGCTCGCATGG | Amplification of the *Hph*-Jun fragment from pKatHPH4 |
| TailHph | TCTTGTGCTGTTGGATCCGTGTTTCAGTTAG (c) |
| TailSacB | TAACTGAAACACGGATCCAACAGCACAAGAG | Amplification of the *SacB*-Jun fragment from pGY13507 |
| DwnXho559 | GACTGGAAAGCGGGCAGTG (c) |
|  | | |
| **Amplification of the fusion fragment (joining PCR procedure)** | | |
| FwdBglII | GGCC**AGATCT**ATCGAATTCGAGCTCGCATGG | Amplification of the fusion fragment including the Hyg^R^ cassette and P*_spac_*::*sacB* from a mixture of overlapping *Hph*-Jun and *SacB*-Jun fragments |
| DwnXho559 | GACTGGAAAGCGGGCAGTG (c) |
|  | | |
| **Construction of IS*Dra2*-104 derivative (strain GY14310)** | | |
| **Amplification of L-Jun fragments** | | |
| GroUP | GCGTCATAGACTCAGATTGTCAG | Amplification of the L-JunZ fragment containing the *tetA* portion and the P*_tnpA_* from genomic DNA of GY13115 |
| TailZ | AGTAACTTCCATCTGGAAAAGCTAGAGGCTTCCGC (c) |
| Tail93 | TAGCTTTTCCAGATGGAAGTTACTGACGTAAGATTAC | Amplification of the Z-JunLE fragment containing *lacZ* from plasmid pGY11556 |
| DwnZPst | TTCG**CTGCAG**GCGGGCAGTGAGAGATCCG (c) |
| **Amplification of the fusion fragment (joining PCR procedure)** | | |
| GroUP | GCGTCATAGACTCAGATTGTCAG | Amplification of the fusion fragment from a mixture of overlapping L-JunZ and Z-JunLE fragments |
| DwnZPst | TTCG**CTGCAG**GCGGGCAGTGAGAGATCCG (c) |
|  | | |
| **Amplification of the Cam^R^ cassette** | | |
| KcamPst | CCGG**CTGCAG**AGCTCGCATGGAGACCGAGG | Amplification of the Cam^R^ cassette from pZT29 |
| KcamHd | CCGC**AAGCTT**CGTTTAAGGGCACCAATAAC (c) |
| **Amplification of the RE-*tetA*” fragment** | | |
| RE138 | CCGG**AAGCTT**TGCGCTGAACATTCGGCGTG | Amplification of a fragment containing RE and the *tetA* portion downstream of the target site from genomic DNA of GY13115 |
| RR | CTCTCCCTTATGCGACTCCT (c) |
|  | | |
| **Diagnostic primers** | | |
|  | |  |
| **Strain GY13109** | |  |
| Tet 729 | GCTTGGTTATGCCGGTACTG | Verification of replacement of the active IS*Dra2* with the Tet^R^ cassette |
| A3 | CCGCCTACTTCGTTATCGAG (c) |
|  | | |
| **Strain GY13111 or GY13115** | | |
| TestC | AGACAATCTGCGCGGATACG | Verification of IS*Dra2*-103 (GY13111) or IS*Dra2*-113 (GY13115) insertion into *tetA* |
| PS93 | TATCCAGCTGAACGGTCTGGTTA (c) |
|  | | |
| **Strain GY13173 or GY13177** | | |
| TetUBam | GAGGTTGCTGATGGACTG | Verification of IS*Dra2*-103Ter116 (or IS*Dra2*-113Term116) insertion into *tetA* |
| Rev116 | GGGGATTTTTTGCAGACG (c) |
|  | | |
| **Strains GY13174 and GY13182** | | |
| 176UP | TTGCAGCGCCTGGGCCTGGACC | Amplification of a fragment encompassing *DR0176* and start of hph gene |
| Hphrev | GGGCGTCGGTTTCCACTATC (c) |
| FwdsacB | CCCAGACCTCCGCCGAATCC | Amplification of a fragment encompassing *sacB* and *DR0179* |
| 179Dwn | GTGGCTGATGGCCTGCTTCTG (c) |
|  | | |
| **Strain GY14310** | | |
| Tet729 | GCTTGGTTATGCCGGTACTG | Amplification of a fragment encompassing the *tetA* portion and *lacZ* gene |
| DwnZPst | TTCG**CTGCAG**GCGGGCAGTGAGAGATCCG (c) |
| LacZout | GATTGGTGGCGACGACTCC | Amplification of a fragment encompassing the Cam^R^ cassette and the downstream *tetA* portion |
| RR | CTCTCCCTTATGCGACTCCT (c) |
|  |  |  |
| **Cloning** | | |
| DraF | CCA**GATATC**ATGACATATGTTATTCTTCCC | Amplification of *tnpA* (cloning into pGY11559) |
| DraX | TAT**CTCGAG**GGTCTCAAGGTTTTTCAG (c) |
| DraF | CCA**GATATC**ATGACATATGTTATTCTTCCC | Amplification of *tnpA* *tnpB* (cloning into pGY11559) |
| DraR | GAA**CTCGAG**TCACACGACTAAAGTCGCGT (c) |
| 1651F | GAGAGCCAG**CATATG**ATAAGGAATAAGGCT | Amplification of the N-terminal portion of *tnpB* (cloning into pGY11559) |
| 1651R | AAGTTCTTGTACGCGGTCTC (c) |
| NdeUptnpA | CCTTTGCCATATGACCTATGTTATTCTTCC | Amplification of *tnpA* with a C-terminal His_6_ tag (cloning into pAPT110) |
| TnpAHISsp | GCTTCCGCATGCTTAATGATGATGATGATGATGTTCCTTATCATGCTGGCTCTCTATGTAT (c) |
| UpNde107 | GTGGAATTGTGAGCGGATA | Amplification of *tnpA* with a C-terminal His_6_ tag (cloning into pGY11559) |
| DntnpAHIS | GCCGGCTCGAGGTTCGCCCAGCTTCTGTATG (c) |
| NdeUPsacB | CCGGCCA**CATATG**AACATCAAAAAGTTTG | Amplification of *sacB* from genomic DNA of *B. subtilis* 168 (cloning into pGY11559) |
| XhoDwnsac | AACGCG**CTCGAG**AATGCCAATAGGATATCGG (c) |
|  | | |
| **PCR amplification to detect insertions into *sacB*** | | |
| CamDwn | TCTGCCGACATGGAAGCCATC | Primer in the *cat* gene |
| RevsacB | GCTGTCTTTGACAACAGATG (c) | Primer at the end of *sacB* CDS |
|  | | |
| **PCR amplification to detect the rejoined donor junction in strain GY13186** | | |
| P1 | GCTTGGTTATGCCGGTACTG | Primer upstream the LE of IS*Dra2*-113Term116 |
| P2 | CTCTCCCTTATGCGACTCCT (c) | Primer downstream the RE of IS*Dra2*-113Term116 |
|  | | |
| **PCR amplification to detect the IS circle junction in strain GY13186 and inverse PCR** | | |
| P3 | ATCTCAAGGGGAAGAATAAC | Primer in LE of IS*Dra2* |
| P4 | TGAACATTCGGCGTGAAGCG (c) | Primer in RE of IS*Dra2* |
|  |  |  |
|  | | |
| **Sequencing** | | |
| UpNde559 | AAGACCGGCCCGATCTACG | Sequencing of inserts cloned into pGY11559 |
| LEext | GGGCTAACTCATGACTGAAG | Sequencing of inverse PCR products |
| REext | ACGCTCATGGAGGCTATGTC |
| Tet729 | GCTTGGTTATGCCGGTACTG | Sequencing of the *lacZ* fusion into GY14310 |
| Zint1 | GTGATGGTGCTGCGTTGGAG |
| Zint2 | GATGAGCGAACGCGTAAC |
| Zint3 | GCTCCACAAGGTAAACAG |

^a^tags with restriction site are in bold. (c) Sequence is on the complementary strand.
